# Supplementary material for: Are carbon emissions trading and green financial instruments synergistic? -Comprehensive quantitative research based on content analysis
Source: PLoS One. 2024 Mar 7;19(3):e0298601. doi: 10.1371/journal.pone.0298601 (PMC10919601; doi:10.1371/journal.pone.0298601)
Supplement: S1 Data — (ZIP) [file pone.0298601.s001.zip › Data/地级市Z_F工作报告.zip!/╒π╜¡/╬┬╓▌╩╨/└┤╫╘╣1⁄2╓┌║┼í╛┬φ┐╦╩2╛▌═°í┐_2017.docx]

一、过去五年及2016年工作回顾

市十二届人大一次会议以来，面对错综复杂的宏观环境和前进道路上的风险挑战，我们在省委、省政府和市委的坚强领导下，凝心聚力、迎难而上，全力打好“拆治归”转型升级组合拳，推动经济发展迈上新台阶、各项社会事业取得新成就。全市地区生产总值年均增长7.6%，突破5000亿元；一般公共预算收入年均增长10.2%；固定资产投资年均增长20.5%，五年累计达到1.5万亿元，是前五年的3.5倍；城镇和农村居民人均可支配收入年均分别增长8.9%、9.9%，城乡收入差距进一步缩小。

五年来，我们着力稳增长促转型，经济运行企稳向好。坚持把化解“两链三险”作为首要任务，累计帮扶风险企业1600多家、处置不良贷款1590亿元，不良率从最高时的4.68%下降到2.69%，房地产市场保持稳定。制定实施“五一〇产业培育提升工程”，持续开展扶工兴贸、驻企服务系列活动，经济运行逐步走出低谷，近两年主要经济指标增幅高于全国全省平均水平。大力推进“四换三名”“三转一市”，新增股份制企业698家，新增境内外上市企业12家，新增高新技术企业518家。先后获得全国质量强市示范城市、国家知识产权示范城市等荣誉。产业集聚区、开发区（工业园区）整合提升步伐加快。成功举办世界温州人大会，温商回归到位资金超4000亿元。现代农业健康发展，农业“两区”建设成效明显。商贸、旅游、金融、电子商务等产业加快发展，服务业增加值比重从46.7%提高到55.4%。

五年来，我们着力强基础优环境，城乡面貌明显改观。进一步完善城市总体规划和土地利用总体规划，洞头实现撤县设区，市区建成区面积扩大到248.7平方公里。实施“温州赶超发展三年超万亿投资计划”，交通、水利投资连续多年全省领先，甬台温高速复线、龙丽温高速等一批重大项目开工建设，金温铁路扩能改造、诸永高速延伸线等工程建成投用。加快推进瓯飞等围垦工程，新增围垦9.8万亩、造地4.3万亩，争取用海指标6万亩，极大地拓展了发展空间。持续开展“五水共治”“三改一拆”，基本消除垃圾河、黑臭河，累计拆违1.2亿平方米，绿化造林56万亩，人均公园绿地面积增加6平方米，“六城联创”圆满收官。市区亮点区块初显形象，各县（市）城区展现新貌。洞头、文成、泰顺创成国家级生态县。完成农村生活污水治理3243个村，建成美丽乡村精品村402个、美丽宜居示范村64个。

五年来，我们着力抓改革促开放，发展活力进一步激发。先后承担了52个国家和省级改革试点。国家金融综合改革取得阶段性成果，农村产权制度改革和“三位一体”合作体系、社会力量办学办医等重点领域改革成效初显。调整完善城建、国资管理体制，创新投融资机制，社会资本参与基础设施建设取得新突破。温州机场、状元岙港区升级为国家一类口岸，新增国际航线24条。保税物流中心（B型）封关运作，口岸通关环境进一步优化。支持企业“走出去”发展，对外投资、境外经贸合作区建设取得新进展。获批海峡两岸（温州）民营经济创新发展示范区。圆满完成援疆、援藏和对口支援工作任务。

五年来，我们着力惠民生增福祉，社会保持和谐稳定。将每年新增财力75%以上用于改善民生。实现县域义务教育基本均衡全覆盖，温州肯恩大学、浙江安防职业技术学院等高校建成招生，温州大学城市学院转设为温州商学院。公立医院改革全面推进。新（扩）建市县骨干医院32家，引进建成民营医院37家。文体惠民工程深入实施，公共文化服务综合评价位次在全省大幅前移，人均体育设施面积显著增加。电视剧《温州一家人》《温州两家人》先后在央视一套热播。社保覆盖面进一步扩大，“五险”参保人数净增237.9万人，养老服务体系初步建立。市区“安置提速”专项行动顺利完成。实施精准扶贫计划，全面消除家庭人均年收入4600元以下贫困现象。积极构建立体化社会治安防控体系，“两抢”案件、命案发案数分别下降89.7%、52.9%。持续开展消防和安全生产专项整治，事故起数和死亡人数分别下降30.9%、24.9%。深化矛盾纠纷排查化解，妥善处置了一批历史遗留问题和信访积案。国防动员和后备力量建设稳步推进，全国双拥模范城创建实现“四连冠”。工会、共青团、妇女、儿童、残疾人、红十字、慈善、老龄事业全面发展，民族、宗教、外侨等工作取得新成绩。

五年来，我们着力转职能优服务，政府自身建设得到新加强。完成两轮政府职能转变和机构改革任务，“五张清单一张网”全面落实，行政审批中介服务市场化改革深入推进，市县两级精简权力事项2/3以上。深入推进法治政府建设，获准行使地方立法权。自觉接受人大法律监督、工作监督和政协民主监督，办理人大代表建议2455件、政协提案2616件。严格落实中央八项规定精神，持之以恒纠“四风”，“三公”经费大幅压减。加强行政监察和审计监督，开展招投标市场专项整治、规范津补贴督查等活动，严肃查处了一批违纪违法案件，政府系统廉政建设取得新成效。

2016年是“十三五”开局之年，也是本届政府任期的最后一年。我们坚持稳中求进的工作总基调，制定实施系列“三年行动计划”，扎实做好补短板各项工作，联动推进经济、城市和社会转型，较好完成了市十二届人大六次会议确定的目标任务。

我们狠抓实体振兴，推动经济发展争先进位。全面落实“三去一降一补”政策措施，实施两化深度融合、领军企业和高成长型企业培育计划，出台企业降本减负“40条”，加大企业帮扶和风险化解力度，经济运行呈现稳中有进、稳中提质的态势。全年实现地区生产总值5045.4亿元，增长8.4%；一般公共预算收入439.9亿元，增长7.7%；固定资产投资3905.7亿元，增长13%；城镇居民人均可支配收入47785元，增长8.5%；农村居民人均可支配收入22985元，增长8.2%。列入省对市经济工作责任督查考核的7项指标，我市综合得分居全省首位。

我们强力推进“大拆大整”，重拳除隐患、治乱象。深刻吸取鹿城双屿农房倒塌的教训，大彻大悟、痛下决心，组织开展八大领域专项整治，全力推动城市治理和产业转型双升级。全年治理城镇危旧房4423幢、农村危旧房9269户；完成城中村改造签约4.6万户、拆除4万户；处置违法建筑2132万平方米；完成“四无”生产经营单位整治12万家，其中关停取缔4.4万家；整治出租房、合用场所18万家，整治市区旧市场32家，实现了“年内初见成效”的阶段性目标。

我们加快产业平台建设，倾力打造发展新引擎。制定实施产业平台整合提升行动计划，初步构建了以温州高新区、温州经开区、产业集聚区为核心的平台布局体系。浙南科技城征迁工作大步推进，创新创业新天地等一批项目落地开工。浙南产业集聚区、瓯江口产业集聚区建设步伐加快，重大产业项目招引取得新进展。生命健康小镇、时尚智造小镇和平阳宠物小镇初展形象。启动小微园建设新三年行动计划，建成小微园550万平方米。实施科技创新“新十条”“人才十条”，新增市级以上众创空间65家，入驻团队2400个，新增国千省千人才22名。实施市区工业企业搬迁改造行动计划，完成企业搬迁1459家。

我们聚焦大项目建设，进一步扩大有效投资。杭温高铁可研报告获批，三澳核电前期取得重大突破，市域铁路S1线、乐清湾铁路支线、绕城高速西南线等加速推进，龙丽温高速文泰段开工建设。重大基础设施、重大产业、高新技术产业、生态保护和环境治理投资，分别增长30.4%、38.4%、19.3%、74.2%。垦造耕地2.7万亩，实施城镇低效用地再开发3.4万亩。积极争取中央预算内资金补助、企业债券发行和专项建设基金支持，与多家金融机构签署合作协议，新增银行授信3200亿元，20个总投资270亿元的政府和社会资本合作（PPP）项目落地。

我们坚持民生需求导向，持续增进人民群众福祉。较好完成年度民生实事项目。全市新增中小学校舍68万平方米，东部基础教育提升三年行动计划扎实起步。分级诊疗全面实施，基层就诊比例提高到60.4%。落实职工医保个人账户家庭“共济使用”政策。公共文化服务体系进一步完善，新建城市书房10家、文化驿站11个、农村文化礼堂164个。开展城市道路综合整治、城市管理治乱、农贸市场改造提升等专项行动，城市序化洁化和文明程度得到新提升。深入推进治水治气，平原河网氨氮和总磷平均浓度分别下降30.7%、12.9%，空气质量优良率达95.4%。市区天然气转换工程加速推进。通用航空应急救援体系启动建设。众志成城做好防台抢险救灾工作，尽最大努力减少灾害损失。圆满完成护航G20杭州峰会维稳安保任务，社会大局保持和谐稳定。

各位代表，过去五年的成绩来之不易。这是省委、省政府和市委正确领导的结果，是全市上下齐心协力、克难攻坚、奋力拼搏的结果。在此，我代表市政府向全市人民和广大建设者，向人大代表、政协委员，各民主党派、工商联、人民团体和社会各界人士，向驻温部队、武警官兵和中央、省驻温单位，向情系家乡的海内外温州人，向所有关心和支持温州发展的港澳台同胞、海外侨胞和国际友人，表示崇高的敬意和衷心的感谢！

我们也清醒地看到，温州经济社会发展中还存在诸多困难和问题，政府工作还有不少差距。对照建设“三个城市”的目标，综合交通、科技创新、产业竞争力和公共服务供给方面的短板还比较突出；人才数量和结构还不适应转型发展、动能转换的需要，吸引高端要素集聚的软硬环境建设有待加强，“两链三险”问题尚未彻底解决；影响发展的体制机制障碍仍然不少，改革创新的锐气需要进一步激扬；城乡规划建设管理水平不高，城市功能和环境品质亟待提升；安全发展的基础还不稳固，治危拆违、防灾减灾、安全生产和消防安全整治任务艰巨，特别是鹿城双屿发生农房倒塌事件，造成重大人员伤亡，令人痛心；人民群众对食品安全、交通出行、教育质量方面的意见相对集中；一些政府工作人员思想观念陈旧、认知水平落后，服务意识、责任意识不强，不作为、不担当，政府职能转变和管理方式还跟不上时代发展变化，依法行政、高效施政的能力需要提高。我们一定要正视矛盾、直面问题，采取更加有效的措施认真加以解决。

二、今后五年目标要求和2017年重点工作

前不久闭幕的市第十二次党代会，描绘了温州未来五年发展的宏伟蓝图，提出了巩固提升温州在全省“铁三角”地位的奋斗目标。我们要正确认识温州所处的历史方位和使命担当，始终保持敢为人先的勇气、改革创新的锐气、蓬勃向上的朝气，谋发展、创新业，抓住机遇、乘势而上，努力再现温州发展的新辉煌。

今后五年政府工作的总体思路是：高举中国特色社会主义伟大旗帜，以邓小平理论、“三个代表”重要思想、科学发展观为指导，深入贯彻习近平总书记系列重要讲话精神和治国理政新理念新思想新战略，按照“五位一体”总体布局和“四个全面”战略布局，全面贯彻落实党中央国务院、省委省政府和市委重大决策部署，持续推进“大拆大整”“大建大美”，着力加快“三大转型”，大步迈向“三个城市”，巩固提升温州在全省的“铁三角”地位，奋力谱写浙江新方位下温州发展的辉煌篇章。

全市经济社会发展的主要目标是：全面完成“十三五”规划，到2021年地区生产总值超7000亿元、年均增长7%以上，一般公共预算收入年均增长7%，固定资产投资年均增长10%以上，研发经费支出占生产总值比重达2.6%，城镇和农村居民人均可支配收入年均增长7.5%以上，完成省下达的节能减排降碳指标。

为巩固提升温州在全省的“铁三角”地位，确保完成五年发展目标，我们要谋划实施“八大攻坚”、实现“八个倍增”：一是开展“大拆大整”“大建大美”攻坚，实现拆旧和建新规模倍增；二是开展基础设施建设攻坚，实现基础设施投资倍增；三是开展招商引资攻坚，实现到位内外资总量倍增；四是开展平台建设攻坚，实现重大产业项目投资倍增；五是开展科技创新攻坚，实现研发经费支出、重点技改投资和高新技术企业数倍增；六是开展企业上市攻坚，实现上市企业数倍增；七是开展精准扶贫攻坚，实现有劳动能力的低收入农户人均年收入倍增；八是开展公共服务补短板攻坚，实现财政民生支出倍增。

为此，我们将深化落实以下工作举措。

（一）强化创新引领，加快产业升级和动能转换。

坚持产业强市、创新强市、人才强市、质量强市、品牌强市，实施企业提升发展“八大工程”，推动存量提质增效、增量快速扩张。大力推进信息化与工业化深度融合、制造业与服务业相互渗透，稳固提升电气、鞋服、泵阀、汽车零部件等传统优势产业在全国的地位。引导企业加大研发投入、优化治理结构、对接多层次资本市场，形成大中小企业活力迸发的新格局。培育发展信息经济、生物医药、清洁能源、高端装备制造、新材料、文化创意等新兴产业，加快发展现代服务业，鼓励发展现代农业、海洋经济、旅游休闲等领域的新业态，着力构建现代产业体系。

充分发挥产业平台在全市转型发展中的示范引领作用，强化政府先行投入，按照“产业引领、创新驱动、产城融合”的要求，加快推进温州高新区（浙南科技城）、温州经开区（浙南产业集聚区）、瓯江口产业集聚区、特色小镇和小微园建设，以局部优化的环境和政策洼地吸引高端要素集聚。加强产业政策和人才政策研究，建立健全“政产学研资介”科技成果转化机制，全力抓好浙东南国家自主创新示范区的创建工作。

（二）纵深推进“大拆大整”“大建大美”，全面提升城市能级和品质。

以不获全胜决不收兵的决心，把“大拆大整”进行到底，完成重点建设区域城中村改造，滚动实施城市有机更新，同步推进“村改居”，彻底消除“半城市化”现象。树立规划引领的理念，加强控规修编和城市设计，坚持做地、储备、出让相衔接，全面推进“大建大美”，优化城市功能和建筑形态，大幅提升城市首位度。深入实施城市建设管理提升三年行动计划，从严治理城市顽疾，推进智慧城市建设，提高城市精细化管理水平。

围绕打造全国性综合交通枢纽城市的定位，全面推进交通“六大工程”，着力形成多元立体、互联互通、安全便捷、绿色智能的现代综合交通体系。坚持以交通圈拓展都市圈、带动经济圈，协同推动城市东拓西优、南连北接、跨江面海发展。坚持市域统筹，加强各县（市）新城区开发和中心城镇建设，优化城镇空间和产业布局，提升温州都市区的整体形象和综合竞争力。

（三）全面深化改革开放，增强发展动力和活力。

以更大的决心和勇气深化改革开放，用创新的思维和举措开辟发展路径，用足用好改革试点政策，为转型发展注入不竭动力。深化金融综合改革，推动金融与产业升级、金融与科技创新紧密结合，更好地发挥“金改”对经济转型的助推器作用。深化投融资体制改革，鼓励民间资本兴办社会事业。开展国家海域综合管理创新试点，统筹推进海洋资源科学利用、海洋生态环境再造和用海管理体制的完善。健全完善功能区、小城镇管理体制和市区财政体制。按照“最多跑一次”的理念和目标，全面深化“放管服”改革，促进政府服务质量和效率双提升。

把握全球化带来的机遇，多渠道扩大对外开放合作，强化实体经济与利用外资的有效结合，进一步优化引资的结构和质量。深入实施优进优出战略，加强海关特殊监管区域、跨境电商产业园等开放平台建设，促进外贸提质增效。主动对接“一带一路”国家战略，支持我市传统优势产业输出产能、赢得市场；鼓励有条件的企业开展跨国并购，高水平建设境外经贸合作区，着力推动温商国际价值链的新布局。

（四）坚持绿色发展，全力打造生态文明先行区。

按照“决不把违法建筑、污泥浊水和脏乱差环境带入全面小康”的要求，扎实开展系列生态创建活动，努力打造美丽中国的温州样本。深化“五水共治”“三改一拆”和小城镇环境综合整治，以治危拆违、截污纳管为重点，统筹推进“水岸同治”，实现“无违建”创建全覆盖，着力补齐生态环境短板。深入实施大气、土壤、近岸海域污染防治计划，加强垃圾分类和减量化管理。调整用能结构，提高资源节约集约利用水平。

积极践行“绿水青山就是金山银山”理念，大力发展生态经济。支持文成、泰顺等生态功能区加快人口跨区域集聚和转产转业，加大生态补偿力度，推动生态强县、绿色富民。加强对乡村建设的规划指导，推动村庄规划和农房设计落地，整体提升美丽乡村建设水平。发展绿色产业，打造绿色城市，倡导绿色生活，形成人人崇尚绿色低碳的社会风尚。

（五）践行以人民为中心的发展思想，持续改善民生福祉。

始终把人民对美好生活的向往作为我们的奋斗目标，多渠道增加民生投入，加快发展教育、文化、卫生、体育、养老等各项社会事业，补齐公共服务有效供给短板，不断提高人民群众的获得感、幸福感。坚持以创新带动创业，以创业带动就业，推动实现更高质量的就业。坚持保基本、兜底线、促公平、可持续，稳步提升城乡社会保障水平。深化中国梦和社会主义核心价值观宣传教育，丰富群众性精神文明创建活动，加强信用体系建设，不断提高全民思想道德素质和社会文明程度。

深化平安温州建设，持之以恒抓好道路交通、消防等重点领域的安全生产整治，加快城乡危旧房治理改造，完成重大地质灾害隐患点村庄避让搬迁和综合整治。加强和创新社会治理，深入推进“网格化管理、组团式服务”，切实增强城乡社区服务功能。全面构建立体化、信息化社会治安防控体系，始终保持对违法犯罪活动的严打高压态势，确保社会和谐稳定。

各位代表，2017年是新一届政府履职的第一年。我们要以更高的标准、更严的要求、更实的作风，只争朝夕、时不我待，全力以赴做好开局之年的各项工作，以优异的成绩迎接党的十九大和省第十四次党代会的胜利召开。今年全市经济社会发展的主要预期目标是：地区生产总值增长7.5-8%，一般公共预算收入增长7%，固定资产投资增长11%，力争研发经费支出占生产总值比重达2%，城镇和农村居民人均可支配收入均增长7.5%。

（一）以“大拆大整”为突破口，推动城市建设上水平。

坚定不移推进“大拆大整”。严格按照时间节点扎实推进八大领域专项整治，确保“上半年大见成效、年底再上新台阶”。6月底前，全市“四无”生产经营单位、居住出租房、民宿和存在隐患的旧市场全部整治到位。完善常态化管控机制，全年处置违法建筑1400万平方米，确保新违建“零增长”。全力打好城中村改造攻坚战，同步推进旧住宅区、旧片区改造提升，完成全市81个行政村、3.1万户签约和旧房拆除。认真抓好农村危旧住房治理改造试点、农民自建房管理体制改革试点，全面完成D级危房和涉及公共安全的C级危房治理。统筹做好“大拆大整”后续管理，妥善解决民生利益相关问题。

精心谋划实施“大建大美”。制定出台“大建大美”实施方案，重点推进市区两级18个片区规划建设。坚持规划引领，强化市区联动，统筹拆后片区控规修编、功能布局、产业重构和项目建设，补齐生活配套、小学幼儿园、停车场等公共服务和市政设施短板。加快推进滨江商务区、中央绿轴、高教新区等亮点区块建设，实质性启动建设三垟城市湿地公园，着手规划建设市民中心、温州美术馆等一批功能性项目。加强城市设计和建筑方案设计，全面提升公用设施、市政道路、绿化养护、城市家具等方面的标准，打造精品建筑、精致街区、精美公园。谋划推进温瑞大道二期和南段快速路、瓯江路东延等46条骨干道路规划建设，有序实施市区18条道路综合整治。启动瓯江和温瑞塘河沿岸环境景观提升工程，开展背街小巷整治，打造城市慢行系统，提高住在温州的生活品质。高水平规划建设瑞安东新产城融合示范带、永嘉三江商务区等城市新区，支持中心城镇完善基础设施和公共配套。统筹做好异地安置，提升非实物安置比例，全市新开工安置房4.5万套、531万平方米，竣工1.2万套、142万平方米。

构建完善基础设施网络体系。开工建设杭温高铁一期，启动市域铁路S1线铺轨，加快建设S2线，同步推进高铁北站与市区连接线等配套路网规划建设，统筹抓好场站及周边物业开发。加快甬台温高速复线、龙丽温高速等项目建设，基本建成绕城高速西南线，力争开工金丽温高速东延线、龙丽温高速文景段。提速推进一批国省道改建项目和跨江桥梁建设。完善市区综合交通网络体系规划，优化温州南部交通设施网络布局，深化市域轨道交通、温武铁路、“南连高速”等项目前期，谋划推进沿海高铁、东部高铁枢纽等项目。加强“四好农村路”建设，实现建制村客车“村村通”。把握全省海港一体化和机场资源整合的机遇，提升温州海港、空港发展水平。加快状元岙二期、大小门岛、乐清湾铁路支线等涉港基础设施和集疏运体系建设，开通国际邮轮航线。建成温州机场T2航站楼。加大水利基础设施建设力度，突出抓好21个“百项千亿防洪排涝工程”，继续推进瓯飞一期、瓯江口浅滩二期等围垦工程，深化瓯江引水项目前期研究。加快建设温州液化天然气（LNG）项目，6月底前建成甬台温、金丽温天然气输气管道。深化泰顺抽水蓄能电站前期工作。科学规划布局新能源汽车充电设施。抓好30个110千伏及以上输变电项目建设。

全面提升城市管理水平。扎实做好全国文明城市、国家卫生城市复评工作，巩固提升“六城联创”成果。启动实施市区一体化发展三年行动计划，推动市辖四区规划共谋共绘、基础设施共建共享、供排水等公共服务同网同标。深化城市治堵，继续推进打卡口、接断路、建环线，优化中心城区至周边县（市）公交线路，启动建设快速公交（BRT）2号、3号线，建成1号线延伸段。积极推进海绵城市和地下综合管廊建设，完成架空管线上改下106公里。加强城市智慧化管理平台建设，实施智慧交通、智慧城管等一批信息惠民工程，启动交通指引、城市照明、水电气等公共设施智能化改造。加快新一代信息基础设施网络建设，基本实现公共场所免费无线网络全覆盖。推进绿色建筑和建筑工业化发展，落实新建住宅全装修政策。加强市容环境卫生和小区物业管理，努力展现整洁有序、文明和谐的城市管理新秩序。

加强城乡环境综合治理。深化“五水共治”，完善落实河长制，6月底前基本消除省控市控劣Ⅴ类水质断面，年底前全面消除劣Ⅴ类水体，努力再现“美丽浙南水乡”风采。加快实施瓯江、飞云江、鳌江流域综合治理，完成市区98个片区截污纳管，建成西片污水处理厂、中心片污水处理厂主体工程。加强农业面源污染治理，实现农村生活污水处理设施常态化运维。深入开展大气污染防治“六大行动”。抓好农田土壤污染治理试点。健全完善餐厨垃圾收集处置体系，建成西向生态填埋场、临江垃圾焚烧发电厂三期。深入实施小城镇环境综合整治，推动115个整治乡镇环境面貌明显改观。实施“蓝色海湾”工程，加快海洋生态整治修复。进一步提高绿化设计管养水平，新建绿道140公里、城市绿地600公顷。

（二）以动能转换为导向，加快产业转型升级步伐。

大力改造提升传统制造业。落实“中国制造2025”和“互联网+”行动计划，坚持以两化深度融合为主抓手，加快推进企业制造装备智能化、生产过程数字化、现场管理精益化，对传统产品更多地植入文化与科技元素，培育两化融合示范试点企业20家、智能制造示范企业10家、上云企业1.2万家。抓好乐清电气、永嘉泵阀、瑞安汽摩配、鹿城鞋革四大省级“机器换人”行业试点，新增机器人应用1000台。扎实推进“三强一制造”，实施制造业和产品质量提升专项行动，新增“浙江制造”认证企业5家。深化“工业创强”行动，提升乐清、瑞安等地工业发展在全省的位次。实施龙头企业培育、中小企业升级工程，主动服务好我市龙头企业的裂变式发展；滚动实施小微企业三年成长计划，新建小微园20个、竣工300万平方米。开展“十百千”助企服务活动，促进惠企政策落地见效，切实减轻企业税费负担。深入推进高耗能、重污染行业整治，淘汰落后产能企业200家。

加快发展现代服务业。培育壮大现代金融、文化创意、电子商务、通用航空、商贸物流等生产性服务业，提升发展10个省级服务业集聚区。完善总部经济政策，大力发展楼宇经济。加大做地储备力度，科学把握供地规模、结构和节奏，完善房地产市场调控长效机制，促进房地产市场平稳健康发展。积极创新服务模式，推动休闲娱乐、健康养老、家政服务等生活性服务业提升发展。开展放心市场、放心餐饮、放心网店创建，助推放心消费在温州。理顺全市旅游发展体制机制，大力推进全域旅游和“旅游+”发展。突出抓好永嘉、文成、苍南全域旅游示范县建设，提升雁荡山、洞头、江心屿等景区开发管理水平，支持楠溪江、刘伯温故里等创建5A级景区，支持苍南矾山、泰顺泗溪创建省级旅游风情小镇。谋划推出十大旅游精品线，做大旅游消费经济，实现旅游总人数超亿人次、总收入超千亿元的目标。

推进农业供给侧改革。启动粮食生产功能区提标改造，守住粮食生产能力和地方粮食储备红线。加强农业集聚发展平台建设，争创省级农业产业集聚区和特色农业强镇。发展农业大数据，推广智慧农业综合服务平台，加快形成以消费为导向的农业供给体系。推进农产品标牌标识建设，加强农业品牌整合和推广，增加绿色优质农产品供给。积极发展生产、供销、信用“三位一体”农民合作组织，完善农合联组织体系。加强对家庭农场、农民合作社、农业龙头企业的扶持，加快培育新型职业农民。完善农业保险政策，扩大参保率。深入开展“一打三整治”行动，继续推进“浙南鱼仓”修复振兴计划。完成第三次全国农业普查工作。抓好美丽宜居示范村建设，全面完成中心村村庄设计，建成美丽乡村风景线10条、精品村100个，力争20个村创成3A级以上景区。大力发展民宿经济、观光农业和森林康养业，培育30个精品民宿示范点。深入实施“强村计划”，减少经济薄弱村888个。探索多元化精准扶贫，实施低收入农户异地搬迁1.7万人，有劳动能力的低收入农户人均年收入增长15%。支持少数民族地区、革命老区和水库库区加快发展。

构筑完善创新创业生态。强化顶层设计和创新基础投入，启动浙东南国家自主创新示范区创建。重视提升我市现有大专院校、科研机构的创新能力，积极引进大院名校共建创新载体，鼓励大学、科研机构服务我市企业。引导企业多渠道增加研发投入，支持在海内外创新资源密集地区建设创新中心，新增高新技术企业170家、科技型中小企业640家、省级企业研究院15家。强化知识产权保护工作，推动100个重大发明专利产业化。积极培育创业投资市场，发挥科技企业贷款风险池的作用，推动创客创新创业协同发展，集中精力打造10家省级以上众创空间。强化政策引才、平台聚才、待遇留才，切实解决好人才子女就学、住房保障等实际问题，全年新增“千人计划”“万人计划”30人，新增在温就业大学生3.5万人。弘扬“工匠精神”，实施“瓯越工匠”三年行动计划，培养高技能人才2万名。加强企业家队伍建设，营造尊重企业家、服务创业者的良好氛围。

做优做强产业平台。高水平规划建设浙南科技城，加快环境打造和招商引智，建成互联网创新创业基地等示范性项目，创建省“千人计划”产业园。以国家高新区指标体系为导向强化对所在地的考核，推动温州高新区在全国全省的排位显著前移。浙南产业集聚区要以做大经济总量为目标，坚持产业功能与城市功能双提升，突出重大产业项目引进，力争主要经济指标增速走在全省产业集聚区前列，冲刺全省第一方阵。瓯江口产业集聚区要坚持产城联动，加快一期24平方公里基础设施建设，争取引进若干个具有引领性的重大产业项目，高品质打造温州现代化城市新区。加快推进省市级特色小镇建设，5个省级创建特色小镇初步呈现主导产业突出、服务功能集成、生态环境优美的微城市形态。以特色小镇的理念和方式改造整合开发区（工业园区）。推进城区工业企业有序向平台搬迁转移，完成搬迁改造1000家。

全力以赴抓项目招引。温商是温州发展最宝贵的财富，要充分发挥温商资源遍布海内外的独特优势，加大回归和对外嫁接力度，推进温州人经济与温州经济协调发展。坚持以大项目、实业项目为主，强化服务、优化政策，内外并举提高引资水平，力争产业项目到位内资650亿元，实际利用外资3亿美元。抓住三澳核电、威马汽车、奔腾激光等项目落地的契机，有重点地培育清洁能源、新能源汽车和激光光电等新兴产业。积极引进和培育以新一代信息技术为核心的新经济、新业态、新模式，加快推动大数据、云计算、移动互联网在城市管理、政府服务、社会治理和智能制造等领域的广泛应用。突出抓好信息软件、传感器、物联网等产业发展，提高对传统产业转型提升的基础支撑能力。

（三）强化要素和制度供给，为转型发展提供保障。

着力优化投资结构。完成固定资产投资4335亿元，确保重大基础设施、重大产业项目、重点技术改造、生态环保等领域投资增长15%以上。强化政府投入和项目引领，稳步推进政府和社会资本合作（PPP）模式，创新社会投资载体，拓展民资进入渠道，力争民间投资占比达到60%以上。统筹安排政府性投资项目建设时序，用足用好各类政府性基金和政策性资金，多措并举降低融资成本。科学推进围填海区域有序开发利用，完成垦造耕地2.2万亩。积极盘活存量土地，实施城镇低效用地再开发2万亩。

进一步提升对外开放水平。充分发挥保税物流中心（B型）的作用，提高指定口岸、商业快件监管中心等开放牌照的综合效益，培育海关高级认证企业和外贸综合服务企业，大力发展保税仓储、跨境电商、服务外包，提升外贸货物在温州口岸进出关比例。推进综合保税区的申报工作，积极争取市场采购贸易方式试点。鼓励发展进口商品贸易，扩大先进技术装备进口。以国家级临空经济区创建为目标推进空港经济区建设。创新民外合作方式，支持有条件的企业“走出去”建立研发机构、生产基地和营销网络，瞄准有先进技术和知名品牌的境外企业开展跨国并购。积极对接长三角一体化建设。以海峡两岸（温州）民营经济创新发展示范区为抓手，吸引更多台商来温投资发展。

科学配置资源要素。综合运用规划、土地、环保、产业政策等手段，深入推进“三去一降一补”。推广以企业分类评价为基础的差别化要素配置机制，加强对新兴产业、创新型企业、技改项目的用地保障。启动新一轮金融综合改革三年行动计划，强化金融服务实体经济的功能，加快地方法人金融机构发展，推动设立证券公司、保险公司、财务公司，力争全年新增贷款750亿元，新增涉农和小微贷款超100亿元。加大金融风险排查和分类处置力度，争取不良率降至2%。大力推动企业上市，争取股份制企业总数超1000家、上市和报会企业总数超30家、新三板挂牌企业总数超80家。积极拓展私募股权、企业债券、融资租赁等多元化融资渠道，新增社会直接融资200亿元。推广责任保险，完善保险参与社会管理的机制。深化农村集体产权制度改革，抓好农房抵押贷款试点，基本完成农村土地承包经营权确权登记颁证工作。

深化体制机制改革和创新。进一步理顺市与区、功能区体制，增强分级决策执行的能力，实现权责匹配、效能提升。建立健全跨行政区域合作协调机制，推动瓯江口与洞头、温瑞平原、南部副中心等区域一体化发展。继续抓好龙港新型城镇化、柳市中小城市综合改革试点工作。推进监察体制改革试点。启动大警制改革，进一步提升警务效能。落实中央深化统计体制改革任务。出台事业单位科研人员离岗创业办法。开展国资集团改组国有资本运营公司试点，鼓励发展混合所有制经济，积极推动市公用集团珊溪公司上市，规范国企自持物业管理。努力创造更加公平有序的市场环境，深入推进社会力量办学办医办体育等改革试点。

（四）以改善民生为重点，全面加强社会建设。

促进教育均衡优质发展。继续抓好教育现代化县（市、区）创建，今年要新创30所省义务教育标准化学校，再培育150所新优质学校。理顺市区两级教育管理体制，加快推进东部基础教育提升三年行动计划。适应全面二孩政策，实施学前教育扩容提升工程，扶持和规范民办幼儿园发展。坚持立德树人，提高教育质量，加快推进小班化教学，持续提升中小学综合评价监测指标和重点高校录取率。加强教师队伍建设，实施乡村教师支持计划。把“双一流”作为温州新一轮高等教育发展的努力方向，支持温州医科大学、温州大学创建省重点高校，支持温州职业技术学院省高职重点校建设，高水平办好温州肯恩大学、温州商学院等一批高等院校。坚持产教融合、工学对接，推进13所中高职院校迁扩建，引导职业教育更好地服务地方发展。

提升医疗卫生发展水平。启动健康温州建设。深化公立医院改革，加快建立现代医院管理制度，支持民营医院发展，努力打造国家区域医疗中心。深入实施“双下沉、两提升”，确保每个县（市）每天有10名副高以上省市三甲医院医生坐诊，县级医疗资源规范化下沉乡镇覆盖率达80%。重视加强城乡社区医疗服务机构建设，启动26个中心镇卫生院医疗服务能力提升工程，加大基层全科医生培养力度，改善群众就近就医条件。建成市中医院新院二期、市七医新院区，投用平阳、苍南县人民医院新院。支持中医药事业加快发展。全面开展县（市、区）公共卫生专业机构规范化建设，进一步普及应急救护知识技能。推进妇幼保健和计生服务资源整合，构建完善妇幼保健机构网络。

加快发展文化体育事业。重视城市文化培育，注重在城市规划建设中融入瓯越文化元素、体现全民健身需求，广泛开展文体惠民活动，基本建成城市社区“15分钟文化圈”“10分钟健身圈”。加强国家历史文化名城保护和利用，传承历史文脉，留住城市记忆。谋划创作一批温州题材文艺精品，加强地方特色戏曲保护和传承。投用奥体中心一期，加快建设奥体中心二期和温州体校新校区。深化文体领域体制机制改革，引导社会资本参与文化建设、体育设施和赛事运营。积极备战全运会、省运会，办好市第十六届运动会、市民文化节等活动。推进全民健身与全民健康深度融合，着力打造国家运动休闲城市。

加强就业和社会保障工作。落实创业扶持政策，办好第三届创博会。积极推进养老保险制度改革，进一步提高基本养老金和基础养老金标准。深化参保精准扩面，力争法定人员基本养老保险参保率达到80%、基本医疗保险参保率达到98%。加快城乡居民和职工大病保险制度整合，探索建立统一的基本医疗保险制度。深入推进全国养老服务业综合改革、全国医养结合试点工作。加大对残疾人就业创业的扶持力度，全面落实残疾人“两项补贴”。完善城乡最低生活保障制度，农村低保标准达到城镇的95%。支持公益和慈善事业发展。改革社会组织管理制度，更好地发挥社会组织、专业社工和志愿者在社会管理中的协同作用。

深化平安温州创建。开展社会治安“固本强基”行动，严密防范和严厉打击各类违法犯罪活动。扎实推进安全生产领域体制机制改革，深化重点领域安全隐患综合整治，流动人口居住信息登记率和居住出租房、民宿整治合格率达到100%。完善以积分制为主体、以电子卡片为载体的居住证制度。实施特种设备智慧监管平台提升行动。积极创建国家级和省级食品安全示范市县，健全食品药品智慧监管体系，强化源头严防、过程严管、风险严控，确保“舌尖上的安全”。加强社会矛盾纠纷排查调处，提升基层社会治理水平。深入开展“同心同行·共建和谐”活动，依法管理宗教事务。健全基层防灾减灾体系，全面实施地质灾害隐患综合治理和“除险安居”三年行动，提升防范抵御自然灾害的能力。

加强双拥优抚安置工作，创新军民深度融合发展机制，大力支持国防和军队建设。以“聚心聚力、共创共享”为目标，进一步团结海内外的温州人共同发展。支持工会、共青团、妇联等人民团体建设，进一步做好老龄、外侨、人防、气象等工作。

经过广泛征集和充分讨论，今年重点办好十方面民生实事。

1．基本消除地质灾害隐患点550个，新（改扩）建避灾安置点400个。

2．加大治霾、治水、治污力度，全面淘汰改造燃煤小锅（窑）炉，完成17座城镇污水处理设施一级A达标排放改造，农村生活垃圾集中收集处理基本实现全覆盖。

3．加大治堵保畅力度，市区新建公共停车泊位5300个，完成30个旧小区停车设施改造，实施40个重要十字路口交通信号智能化改造；市区新增（优化）公交线路15条，新（改）建公交候车廊100个，30条线路350辆公交车实现移动支付。

4．强化餐桌安全管理，市区新（改）建农贸市场60个，全市建设食用农产品电子追溯农贸市场20个，创建“在线阳光厨房”100个，农产品质量安全抽检合格率稳定在98%以上。

5．建立身份智能识别系统，免费为1万名失智老人、孤寡老人等发放防走失“安心手环”，为7.5万户空巢老人、分散养老特困人员住所安装独立式火灾探测报警器。

6．新建农村居家养老服务照料中心630家。

7．全市新（扩）建70所幼儿园，普惠性幼儿园覆盖面达到85%，等级幼儿园创建率达90%以上。

8．推广家庭医生签约服务，争取户籍人口规范签约率达到30%；完成妇女“两癌”免费筛查12万名。

9．推进文化惠民，新增农村文化礼堂150个，新建城市书房6家，建成文化驿站10个。

10．全市新建标准篮球场50个，建设公园、社区健身步道20公里，市区新建百姓健身房50个。

三、全面提升政府履职能力和水平

新一届政府要按照服务政府、责任政府、法治政府、廉洁政府的要求，以改革创新精神加强自身建设，认真行使好人民赋予的权力，对人民负责、受人民监督、让人民满意。

（一）加强政府行政能力建设。好学是善政之本，只有善于学习、顺应趋势、拥抱变革，才能成为善为政府。我们要全力打造学习型政府，引导广大干部特别是领导干部主动转变观念、提升认知、拓宽视野，加强和改进调查研究，不断提升推动转型发展的能力和水平。我们要加强政府执行力建设，建立健全市级部门和县（市、区）政府重点工作清单制度，确保中央、省市委重大决策部署落到实处。我们要切实改进政风，深化效能建设，开展政府部门效能评估，坚决整治不担当、不作为和“中梗阻”现象，推动政务服务提速提效。再伟大的情怀，不落地，终究是一纸空谈，全体政府工作人员要以争当铁军“排头兵”的决心和勇气，以有为政府的担当、“拼命三郎”的干劲，狠抓各项决策部署落地见效，干在实处、走在前列、勇立潮头。

（二）全面提高依法行政水平。牢固树立职权法定观念，坚持法无授权不可为、法定职责必须为。完善政府立法工作机制，增强立法工作的主动性和有效性。健全依法决策机制，积极推动社会公众参与重大决策。根据新形势新任务及时调整优化政策导向，强化政策执行监督，秉承契约精神，打造诚信政府。完善行政综合执法体制，严格规范公正文明执法，推行行政执法公示制度、执法全过程记录制度、重大执法决定法制审核制度，加强市县两级行政处罚系统建设。积极推进行政复议体制改革。深入实施“七五”普法规划。自觉接受人大及其常委会的法律监督和工作监督，自觉接受人民政协的民主监督，高质量做好建议、提案办理。认真听取民主党派、工商联和人民团体的意见建议。

（三）创新政府服务管理方式。按照群众和企业到政府办事“最多跑一次”的理念和目标，深化“五张清单一张网”改革，积极运用“互联网+”政务模式，年底前行政审批“最多跑一次”事项覆盖80%以上。充分发挥大数据中心的作用，统筹数据共享和开放，解决“信息孤岛”问题。推动办事大厅与网上审批线上线下对接，更多地变“群众奔波”为“信息跑腿”。全面推进行政服务中心“一窗受理、集成服务”改革。深化投资准入制度和便利化改革，增加市场自主选择权，优化审批流程，实现基建项目全流程100天、企业投资审批50天。推广重大投资项目代办制，实现“一次受理、全程帮办”。制定中介服务行业标准和考评机制，完善涉批中介服务公开选取竞争机制。深化商事制度改革，调整监管重心，强化事中事后监管。

（四）加强政府系统廉政建设。始终把纪律和规矩挺在前面，深入贯彻落实中央八项规定、省委“28条办法”和“六个严禁”等正风肃纪各项要求，坚决贯彻党内政治生活准则和党内监督条例，坚决落实廉洁自律准则和纪律处分条例。严格执行财经管理规定，严防各种“四风”新问题。认真履行“一岗双责”，聚焦权力运行风险点，探索“制度+科技”的制约监督机制。强化审计监督，实现公共资金、国有资产、国有资源和领导干部经济责任审计全覆盖。推行作风巡查常态化，从严查处群众身边的腐败问题和不正之风，进一步树立干净干事、清正廉洁的政府形象。

温州正处于转型发展继往开来的关键时期，我们肩负着时代赋予的光荣使命。志不求易者成，事不避难者进。让我们更加紧密团结在以习近平同志为核心的党中央周围，在中共温州市委的坚强领导下，上下同欲，克难奋进，扎实苦干，为巩固提升温州在全省的“铁三角”地位，谱写浙江新方位下温州发展的辉煌篇章而努力奋斗！
